# Characterization and parameterization of aerosol cloud condensation nuclei activation under different pollution conditions

**DOI:** 10.1038/srep24497

**Published:** 2016-04-14

**Authors:** H. C. Che, X. Y. Zhang, Y. Q. Wang, L. Zhang, X. J. Shen, Y. M. Zhang, Q. L. Ma, J. Y. Sun, Y. W. Zhang, T. T. Wang

**Affiliations:** 1 Key Laboratory of Atmospheric Chemistry of CMA, Institute of Atmospheric Composition, Chinese Academy of Meteorological Sciences, Beijing 100081, China; 2 College of Earth Science, University of Chinese Academy of Sciences, Beijing 100049, China; 3 LinAn Regional Atmosphere Background Station, LinAn 311307, China; 4 State Key Laboratory of Cryospheric Sciences, Cold and Arid Region Environmental and Engineering Research Institute, Chinese Academy of Sciences, Lanzhou 730000, China; 5 Trinity Consultants, INC., China office, Hangzhou 310012, China; 6 Heilongjiang Meteorological Bureau, Harbin 150001, China

## Abstract

To better understand the cloud condensation nuclei (CCN) activation capacity of aerosol particles in different pollution conditions, a long-term field experiment was carried out at a regional GAW (Global Atmosphere Watch) station in the Yangtze River Delta area of China. The homogeneity of aerosol particles was the highest in clean weather, with the highest active fraction of all the weather types. For pollution with the same visibility, the residual aerosol particles in higher relative humidity weather conditions were more externally mixed and heterogeneous, with a lower hygroscopic capacity. The hygroscopic capacity (*κ*) of organic aerosols can be classified into 0.1 and 0.2 in different weather types. The particles at ~150 nm were easily activated in haze weather conditions. For CCN predictions, the bulk chemical composition method was closer to observations at low supersaturations (≤0.1%), whereas when the supersaturation was ≥0.2%, the size-resolved chemical composition method was more accurate. As for the mixing state of the aerosol particles, in haze, heavy haze, and severe haze weather conditions CCN predictions based on the internal mixing assumption were robust, whereas for other weather conditions, predictions based on the external mixing assumption were more accurate.

Cloud condensation nuclei (CCN) are the aerosol particles that enable the condensation of water vapor and formation of cloud droplets when subjected to a given supersaturation (SS). Variations of CCN can influence the microphysical properties of clouds, cloud lifetime, precipitation, the hydrological cycle, and climate^1^,^2^,^3^,^4^. As CCN are an essential input for the formation of cloud droplets, the relationship between aerosol and CCN has received increasing attention^5^,^6^, especially for accurate CCN concentration predictions under polluted conditions including haze and fog.

The ability of CCN to activate cloud droplets at a given supersaturation depends on the particle size, chemical composition, and mixing state. The particle size has been reported to be the most important factor in CCN activation and prediction (most of these reports were obtained in relative clean episodes in Europe and N. America)^7^,^8^. However, the chemical composition maybe also important in, for example, marine and polluted environments, where the aerosol chemical composition is quite different to the clean continental environment^9^,^10^,^11^, particularly at relatively low supersaturations^12^,^13^,^14^. In CCN predictions, the particles internal mixing assumption generally overestimates the CCN concentration^15^,^16^, whereas the external mixing assumption underestimates it^16^,^17^. These two mixing assumptions can result in up to ~40% relative error in CCN predictions, thus, the aerosol mixing state definitely needs to be taken into consideration in CCN predictions, especially in places where anthropogenic aerosol emissions are strong and pollution is heavy^16^,^18^,^19^.

The aerosol chemical composition and mixing state can influence the CCN activation ability through changes in the hygroscopic capacity of the particles. The hygroscopic capacity is quantitatively represented by hygroscopicity parameter (*κ*) to link the CCN activity and hygroscopic ability of aerosol particles, and is widely used in CCN predictions^16^,^20^. In recent years, numerous studies have parameterized CCN concentration using aerosol-determined *κ*^11^,^13^,^21^,^22^,^23^. However, observation and parameterization for aerosol CCN activation in China is still rare.

The YRD region is one of the major haze areas in China^24^ and has a high economic growth rate and high population density. The aerosol pollution-induced haze and fog episodes have become increasingly worse in recent years^25^,^26^. In 2013 (the year of this study), the YRD region suffered the most persistent haze and fog events in half a century^27^. The aerosol chemical composition shows unique characteristics between the haze- and fog-polluted environments^28^, and the mixing state may be different to the clean areas; therefore measurements of aerosol CCN activation and CCN concentration predictions are especially needed for the high polluted conditions.

In this paper we present long-term *in situ* aerosol CCN activation measurements, including all four seasons, from January to October 2013, at a regional GAW (Global Atmospheric Watch) station in the YRD. The aerosol chemical concentration and size distribution were also obtained simultaneously during the measurements. The role of aerosol chemical composition, aerosol mixing states, and hygroscopic capacity on activation properties, and their influence under different pollution conditions, especially heavy pollution condition, are investigated, separately. Finally, parameterization of time dependent CCN predictions using different approaches with aerosol size, chemical composition and mixing states under different pollution conditions are also presented and discussed.

## Results and Discussion

### Characterization of CCN activation under different weather-pollution conditions

#### Variation in CCN during two clean-to-polluted episodes

**Figure 1:**
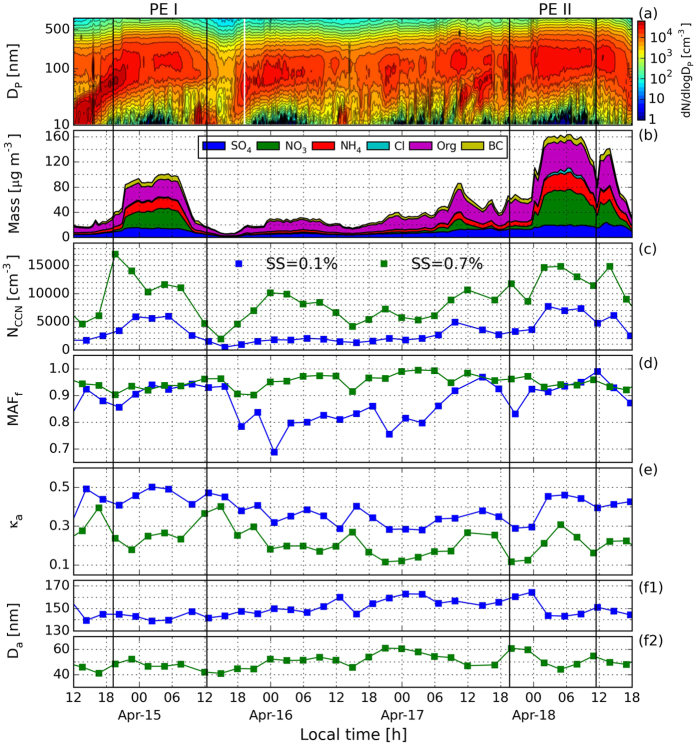
Temporal series of (**a**) aerosol number size distribution, (**b**) AMS-determined components mass concentration, (**c**) CCN number concentration at SS = 0.1% and 0.7%, (**d**) MAF_f_ for both SS = 0.1% and 0.7%, (**e**) *κ*_a_ at SS = 0.1% and 0.7%, (**f1**) critical active diameter at SS = 0.1%, (**f2**) critical active diameter at SS = 0.2%, from clean to polluted.

In Fig. 1a, a new particle formation (NPF) event can be seen around 12:00 on 14 April, with increasing number concentration and increasing size of newly generated particles. (The NPF event during the experiment can also be found in the paper by Shen, *et al.*^29^). After several hours of the event, the number concentration of aerosol particles (mostly fine particles) reached a peak value (~19,000 cm^−3^), and the aged aerosol particles remained at a high level with little change until 12:00 the next day. This was accompanied by a large increase in aerosol chemical components mass concentrations (Fig. 1b), especially for nitrate and organics, followed by ammonium and sulfate. The total aerosol chemical composition mass concentration measured by the AMS increased by nearly four times in the polluted episode compared with clean conditions. This kind of aerosol cycle event occurred several more times, and an almost identical process, starting from 12:00 on 17 April, can be seen in Fig. 1.

The CCN concentration increased during the aged and polluted episodes I and II (marked as PE I and II in Fig. 1) as showed in Fig. 1c. At the end of NPF event (the particle growth period), the CCN concentration at 0.7% SS reached 17,000 cm^−3^ at 19:00 on 14 April. However, the CCN concentration at 0.1% SS only increased in the aged period, which indicated that NPF events can only increase the CCN concentration at high supersaturations. The increased maximum active fraction (MAF_f_, detailed in CCN activation data fitting section) at low supersaturation (0.1%) (Fig. 1d) during the PE I and II episodes also indicated that a large proportion of CCN active particles existed in polluted weather. However, the MAF_f_ at higher supersaturation (0.7%) showed no significant change during pollution, indicating that easily activated particles were mainly in the accumulative mode. In Fig. 1e, the effective hygroscopicity of the aerosols, *κ*_a_ (detailed in CCN activation data fitting section), was relatively higher during the aged and polluted episodes, and was more obvious at lower supersaturation (0.1%),which could be the result of the increase in the proportion of nitrate and other inorganic components during the polluted episode (Fig. 1b).

The variation in *κ*_a_ was opposite to the critical diameter of CCN active particles *D*_a_ (detailed in CCN activation data fitting section). During the polluted episode, the *D*_a_ at both 0.1 and 0.7% supersaturation shifted to smaller diameters, and the variation in *D*_a_ at low supersaturation (0.1%) was more obvious (Fig. 1f1,f2), indicating that particles were more hygroscopic and more easily activated as CCN in the polluted episode, especially larger particles. All these findings suggest that there are large differences in CCN activation abilities in polluted and clean episodes.

#### Classification of CCN activation measurements

**Figure 2:**
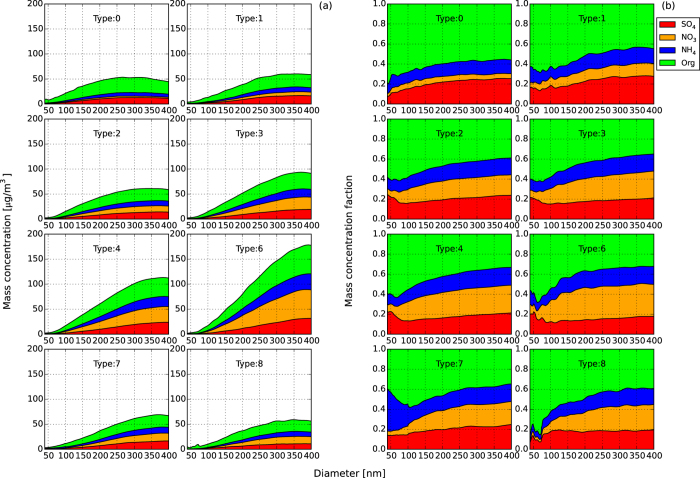
The mean size distribution of non-refractory species (determined by AMS) in terms of (**a**) mass concentration and (**b**) mass concentration fraction under different weather types.

During the measurement period, pollution episodes like PE I and PE II happened frequently. To separate the CCN activation capabilities under different pollution conditions, nine types of weather-pollution phenomena were classified into clean, haze, mist, and fog conditions, corresponding to different extents of aerosol pollution, associating with mean relative humidity (RH) and visibility during each CCNC measurement cycle (Table 1). The classification is based generally on the WMO definition of haze, mist, and fog. The corresponding size-resolved aerosol non-refractory species mass concentration and mass concentration fraction measured by the AMS under different weather types can be seen in Fig. 2.

From Table 1, it can be seen that clean weather conditions with low RH and high visibility (type 0) happened most frequently during the CCN measurement period, accounting for ~30% of the total. This was followed by haze and heavy haze weather (types 2 and 4, respectively), accounting for ~23% of the total, and then fog and heavy fog weather (types 7 and 8, respectively), accounting for ~11%. These weather types were the most common during the measurement period. During the continuous observation period, no CCN spectra were measured under type 5 (transition from mist to fog), which may indicate that transition time from mist to fog was relatively short, probably <30 min of one CCN spectrum scan in this study. In type 7 and 8 weather episodes, the observed aerosol particles were those residual particles, since many CCN active aerosols have been activated into the cloud (fog) droplets which normally have diameters larger than 10 μm, and therefore could not be collected by the inlet of our system^30^.

**Figure 3:**
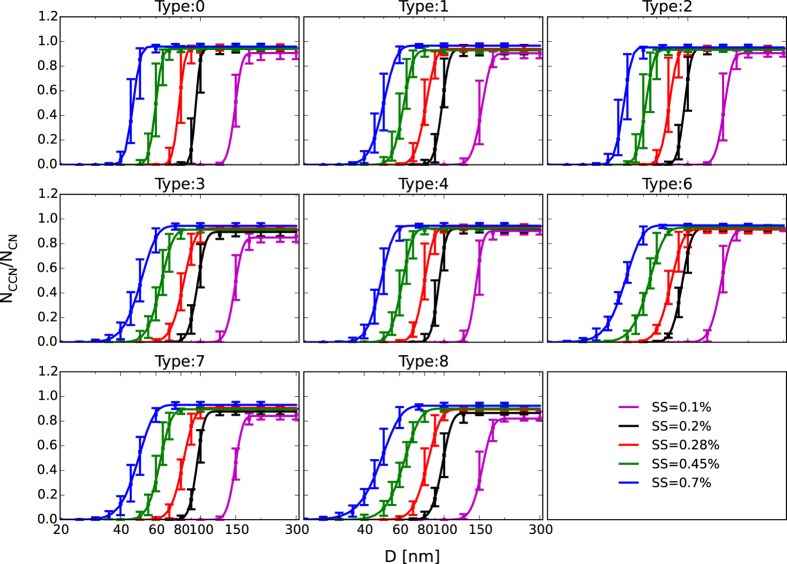
Averaged CCN activation spectra under different weather-pollution conditions. The data points are the median value of all measured spectra during the weather type, the lower and upper bars represent the quartile error, and the lines are derived from three-parameter CDF (Gaussian distribution function) fitting.

The lowest mass concentrations for each chemical component were all found in type 0 weather conditions (Fig. 2a), with a peak concentration of ~50 μg m^−3^ at 300 nm. From clean to severe haze weather episodes, i.e., from type 0 to type 6, the size-resolved aerosol particle mass concentrations and their peak increased progressively (Fig. 2a), accompanied by mode diameters moving to a larger size, and an increase of inorganic aerosol concentration mass fraction (Fig. 3b). The highest total mass concentration value was found during a type 6 episode, with a peak value around 190 μg m^−3^, almost four times higher than a type 0 episode. The largest changes in mass concentration (Fig. 2a) and mass concentration proportion (Fig. 2b) were found for 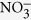, which increased from ~3 to 60 μg m^−3^ and from 5 to 30% from type 0 to type 6.

During the type 7 and 8 conditions (fog and heavy fog episodes, respectively), the size-resolved mass concentration was similar to type 1 conditions (mist) with a larger 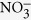 proportion. This probably indicates that fog episodes in the YRD region, which form in polluted conditions, are quite different from those in clean areas.

#### CCN activation curves and heterogeneity of chemical components

The averaged CCN activation spectra under different weather conditions are illustrated in Fig. 3. The CCN activation curve in type 0 (clean) weather was close to the ideal shape at all measured supersaturations, with the sharpest slope of the curves and a maximum CCN active fraction (MAF_f_) close to 1, indicating that aerosol particles in clean episodes were mostly internally mixed with a homogeneous chemical composition.

A flatter CCN activation curve was the most remarkable characteristic in type 8 and type 6 relative to type 0 conditions, showing increasing heterogeneity in particle chemical compositions and increasing proportion of externally mixed particles^11^ with worsening pollution. The difference in CCN activation curves under different weather episodes again indicated that haze and fog pollution can impact CCN activation and further impact cloud and climate.

**Figure 4:**
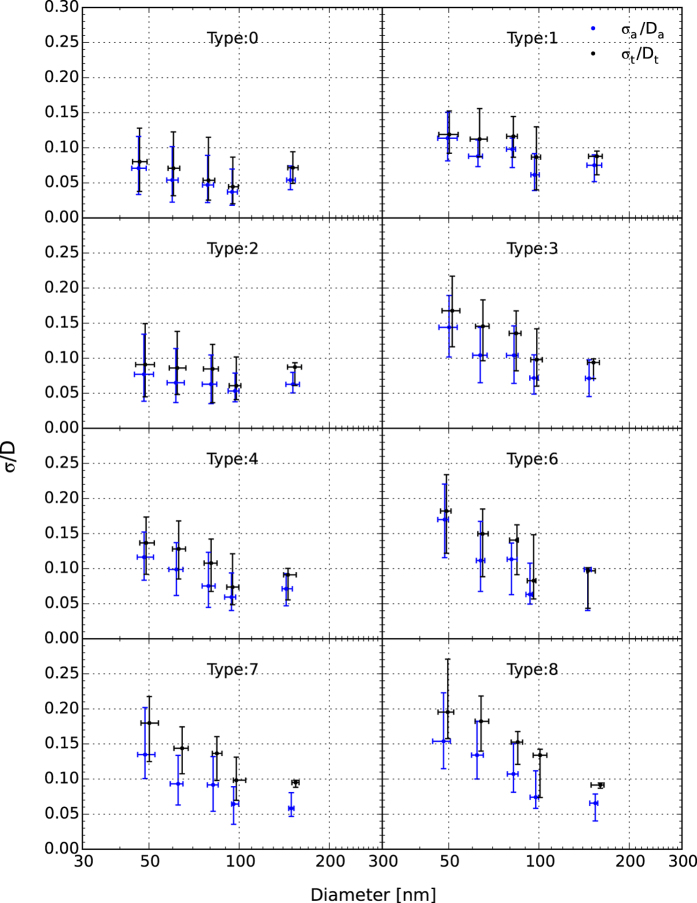
The averaged heterogeneous parameters (*σ*_a_/*D*_a_, *σ*_t_/*D*_t_) derived from CCN activation spectra under different weather-pollution types plotted against the critical diameter (*D*_a_ or *D*_t_, respectively). The data points are the median value corresponding to the weather-pollution type and given supersaturation, and the lower and upper bars represent the quartile error.

The heterogeneity of the aerosol particle chemical components can be inferred from the variation in *σ*_a_/*D*_a_ and *σ*_t_/*D*_t_ (the ratio of the fitting standard deviation and its corresponding critical active diameter, detailed in CCN activation data fitting section) as shown in Fig. 4. *σ*_a_/*D*_a_ and *σ*_t_/*D*_t_ characterize the degree of heterogeneity of CCN active particles at ~*D*_a_ and the overall degree of heterogeneity including CCN active and inactive particles at ~*D*_t_, respectively^11^. From the type 0 (clean period) to type 6 (polluted condition) episodes, both *σ*_a_/*D*_a_ and *σ*_t_/*D*_t_ increased with the aggravation of pollution, suggesting both enhancement of aerosol heterogeneity and external mixing along with worsening pollution conditions. For pollution with the same visibility level, the particles under higher RH weather conditions were more heterogeneous at the same diameters, i.e., the particles with the same diameters in mist (type 1) or heavy mist (type 3) episodes were more heterogeneous than in haze (type 2) or heavy haze (type 4) episodes, which was probably because the internally mixed particles took up water and grew to droplets or large particles and the rest at investigated diameters were generally externally mixed at high RH conditions.

For 45–100 nm size particles, *σ*_a_/*D*_a_ and *σ*_t_/*D*_t_ decreased as the aerosol particles grew for all weather types, showing that particles become more internally mixed with homogeneous chemical composition through nucleation and coagulation processes and so on when growing during the Aitken mode. The values of *σ*_a_/*D*_a_ and *σ*_t_/*D*_t_ at ~150 nm were relatively stable, only varying in the range of 0.05–0.10 in all episodes. For mist and heavy mist weather (types 1 and 3), the heterogeneous parameters at ~150 nm generally remained the same as that at ~100 nm, especially for *σ*_t_/*D*_t_, while for the haze, heavy haze, and severe haze weather (types 2, 4, and 6), both *σ*_a_/*D*_a_ and *σ*_t_/*D*_t_ increased from ~100 to ~150 nm. This may indicate that the mixing state of particles in the accumulation mode was less influenced by the weather, whereas in the Aitken mode, the particles were more heterogeneous in high RH weather conditions, which may also because the fine particles at high RH weather were externally mixed.

During the fog episodes (types 7 and 8), all *σ*_a_/*D*_a_ and *σ*_t_/*D*_t_ values were higher than those during types 0 to 6, indicating that the residual aerosol particles in fog days were more heterogeneous and externally mixed, with low CCN activation abilities.

#### Maximum active fraction (MAF_f_)

**Figure 5:**
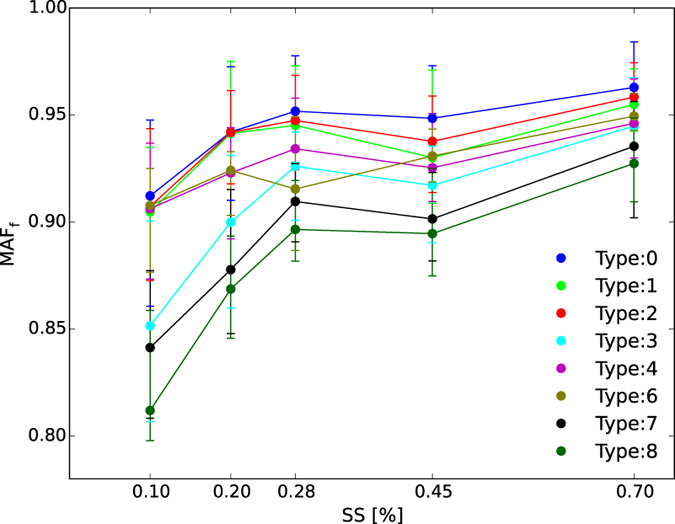
The averaged MAF_f_ derived from three-parameter CDF of CCN activation spectra at different weather-pollution types plotted against supersaturation. The data points are the median value of all MAF_f_ corresponding to the type and supersaturation, and the lower and upper bars represent the quartile error.

The comparison of MAF_f_ at five supersaturations during different weather conditions are presented in Fig. 5. The MAF_f_ in type 0 (clean) conditions was the highest at all measured supersaturations, as expected. A value around 0.9 in lower supersaturation conditions (0.1%), indicated that about 10% of the aerosol particles in the diameter range ~150–250 nm were CCN inactive particles, as the 1-MAF_f_ represents the inactive particle fraction^11^. The MAF_f_ during type 7 and type 8 (fog and heavy fog) conditions were the lowest and second lowest at all supersaturations, respectively, and the lowest value (~0.8) at 0.1% supersaturation was found during heavy fog in this study (Fig. 5), indicating that a larger proportion of hydrophobic (~20%) particles existed at 150–200 nm under high RH.

For different pollution episodes with the same visibility levels, the MAF_f_ under high RH was lower than under low RH at the five supersaturations, which was the same as the variation in heterogeneous parameters, i.e., the MAF_f_ during mist (type 1) or heavy mist (type 3) episodes were lower than during haze (type 2) or heavy haze (type 4) episodes at the five supersaturations. This may because all these episodes with high RH were associated with abundant water vapor available to form cloud (fog) droplets, and only aerosol particles with a large proportion of insoluble chemical components and CCN inactive particles remain in these types of weather conditions, which could reduce the MAF_f_. This phenomenon was more obvious in fog episodes (types 7 and 8) under low supersaturations.

**Figure 6:**
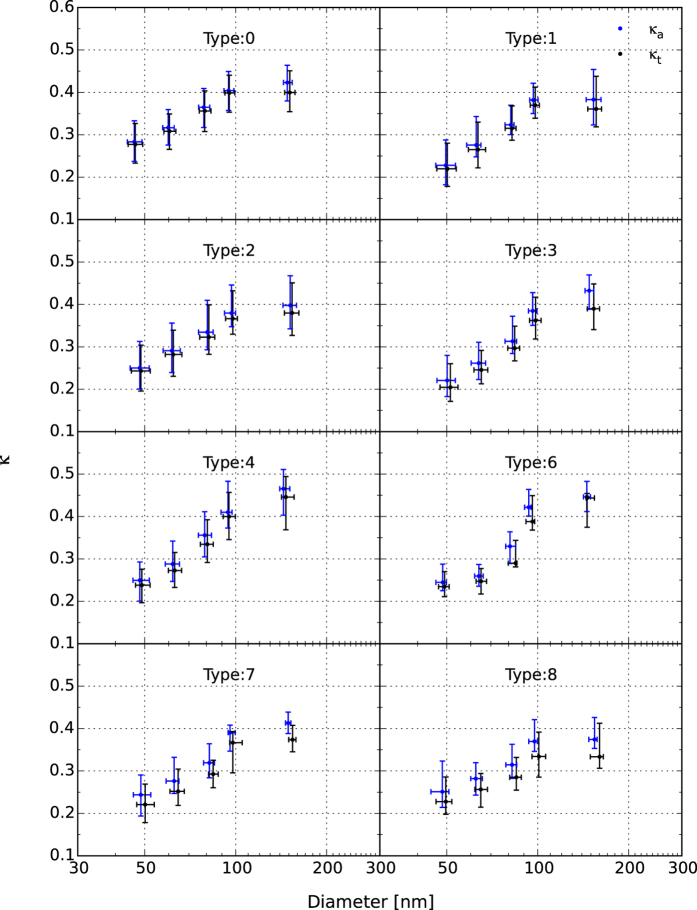
The averaged hygroscopic parameters (*κ*_a_, *κ*_t_) derived from CCN activation spectra under different weather-pollution types plotted against the critical diameter (*D*_a_ or *D*_t_, respectively). The data points are the median values corresponding to the weather-pollution type and given supersaturation, and the lower and upper bars represent the quartile error.

Hygroscopic ability  The change in the hygroscopicity parameter *κ* with increasing aerosol particle size during different weather conditions is shown in Fig. 6. Both *κ*_a_ and *κ*_t_ (detailed in CCN activation data fitting section) increased with increasing particle size and the highest values were found at ~150 nm during all conditions, reflecting that larger particles were more hygroscopic.

*κ*_a_ and *κ*_t_ represent the hygroscopic abilities of CCN active particles and all particles (CCN active and inactive), respectively. As illustrated in Fig. 6, *κ*_*a*_ and *κ*_*t*_ were close to each other in the relatively clean episodes (types 0, 1, and 2), indicating a low fraction of CCN inactive particles. The difference between *κ*_a_ and *κ*_t_ increased with worsening pollution and increasing RH, and finally reached a maximum in type 8 (heavy fog episodes), indicating again that the CCN inactive particles increased at investigated diameters (25–200 nm) under relatively high RH and higher aerosol concentration conditions.

For different pollution episodes with the same visibility level, a similar conclusion was found that both *κ*_a_ and *κ*_t_ were lower under high RH episodes, i.e, *κ*_a_ and *κ*_t_ under mist (type 1) or heavy mist (type 3) episodes were lower than under haze (type 2) or heavy haze (type 4) episodes at all measured diameters. This phenomenon can also be interpreted as the remaining hydrophobic particles in the atmosphere at high RH weather episodes.

The highest *κ*_a_ and *κ*_t_ (~0.45) were found at diameters of ~150 nm in heavy haze episodes (type 4), which was slightly above the value in severe haze episodes (type 6). This can be interpreted in terms of the large increase in the proportion of 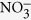, the slight increase in 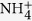, and the decrease in the organic fraction during types 4 and 6 relative to type 0 (Fig. 2b). It also suggests that the aerosol particles were easily activated in heavy or severe haze episodes. The lowest *κ*_a_ and *κ*_t_ at ~150 nm were found in type 8 (heavy fog) as expected.

#### Parameterization of aerosol CCN prediction

The activation of CCN depends on the particle diameter, chemical composition, and mixing state^31^. The particle diameter has been proved to play the main role in CCN activation^8^,^32^. However, on the basis of long-term measurements in continental rural environments in the YRD region of China, the chemical composition of aerosols was found to have a great influence on CCN activity, especially under low supersaturation conditions. Based on this fact, the CCN predictions in this study used particle diameter as the variable, then estimated the effect of particle chemical composition and mixing state on CCN activation.

#### Parameterization using experimental average hygroscopicity (AH)

This was a simplified calculation method. The aerosol particles were classified into nine categories according the weather episode types (Table 1), and a constant value for each category (measured averaged *κ*_t_) was used to represent the particle chemical composition and mixing state. However, it only represents the regional CCN activation due to the *κ*_t_ only represents the aerosol hygroscopicity in YRD.

#### Parameterization using chemical composition variables

In this parameterization scheme, both AMS-derived aerosol particle chemical composition and particles size distribution were considered as variables and the mixing state of particles were assumed to be either internally or externally mixed. The following four methods were used.

- Bulk chemical composition and internally mixed (BI).
- The particle chemical composition was assumed to be size independent and internally mixed in this method, i.e., all particles have identical chemical composition in the entire size range, and the averaged chemical composition was derived from the bulk mass concentration of species measured by AMS and MAAP.
- Bulk chemical composition and externally mixed (BE).
- In this method, the particle chemical compositions was assumed to be size independent as in BI; however, the particles were assumed to be externally mixed in the entire size range, i.e., there were four types of particles at each size: (NH_4_)_2_SO_4_, NH_4_NO_3_, organics, and black carbon, and the concentrations of these four types of particle at each size were identical.
- Size-resolved chemical composition and internally mixed (SI).
- The aerosol particle chemical compositions were considered to be varied in the entire size range in this method. At each particle size, the particles were assumed to be internally mixed with identical compositions. The compositions were derived from the mass size distribution measured by AMS. It is noteworthy that due to the measured error of size-resolved mass concentration by AMS, in the CCN predictions with size-resolved chemical concentration, the aerosols were divided into two categories: inorganic and organic. In this method, those two categories of particle were internally mixed.
- Size-resolved chemical composition and externally mixed (SE).

The particle chemical compositions were considered to be varied in the entire size range; however, the particles were assumed to be externally mixed in this method. At each particle size, there were two types of aerosol particles: inorganic and organic.

The predicted CCN number concentrations were calculated using the method in CCN prediction section. The *κ* of aerosol inorganic species were known^33^; however, *κ* for organic particles was uncertain due to the complexity of organic aerosols. To investigate the general hygroscopic ability of organic aerosols in the YRD region, the observed effective *κ*_a_ was fitted with the measured organic volume fraction, and the data where mass concentration <1 μg m^−3^ were excluded to reduce the error^15^.

**Figure 7:**
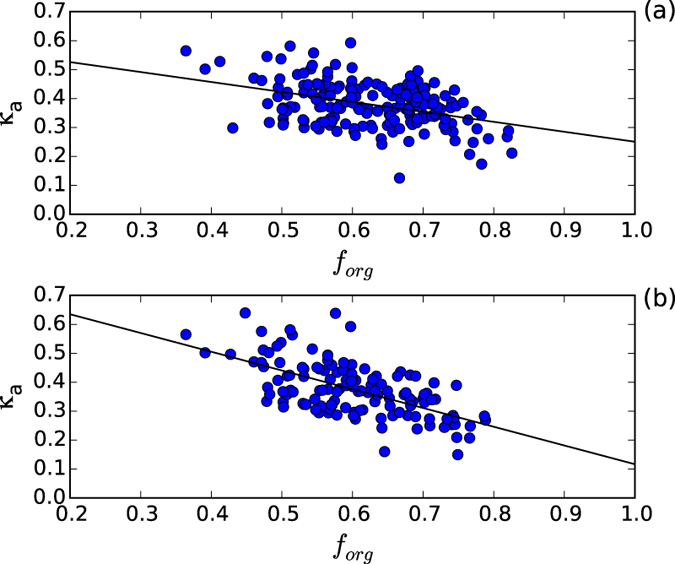
Correlation between the observed effective hygroscopicity parameter of CCN active particles (*κ*_a_) and the organic volume fraction (*f*_org_) during weather (**a**) type 0, 1, 3, 7, and 8 and (**b**) type 2, 4, and 6.

As illustrated in Fig. 7, the correlation between *κ*_a_ and the organic volume fraction can divide into two categories. Category 1 (Fig. 7a) represents the correlation under weather types 0, 1, 3, 7, and 8 (clean, mist, heavy mist, fog, and heavy fog episodes), where *κ*_org_ = 0.2 and *κ*_inorg_ = 0.6. Category 2 (Fig. 7b) represents the correlation under weather types 2, 4, and 6 (haze, heavy haze, and severe haze episodes), where *κ*_org_ = 0.1 and *κ*_inorg_ = 0.7.

The result shows that the hydrophilic organic chemical component fraction decreased in haze pollution, which is different to a previous study in Hong Kong^19^. For the hygroscopic capability of inorganic aerosols, in haze weather episodes, *κ*_inorg_ was higher than in mist weather, which can be explained by the large increase in highly hygroscopic particles such as nitrate (Fig. 2b). The obtained *κ* values of both organic and inorganic aerosols under clean and mist and fog episodes were similar to observations in North America and the Amazon region^13^,^34^,^35^.

The fitting result of predicted and measured CCN number concentration is illustrated in Table 2. From Table 2, it can be seen that the external mixing methods were more accurate for the fog and clean episodes (types 7, 8, and 0) at all five supersaturations. This may be because the internally mixed particles with homogeneous composition had already hygroscopically grown into larger particles or were active as cloud droplets under high RH environments. Simultaneously, heterogeneous reactions may have existed in the fine particle formation and the remaining particles were mostly externally mixed. As for the clean episodes, the low concentration and simple species made the external mixing method better for CCN prediction.

**Figure 8:**
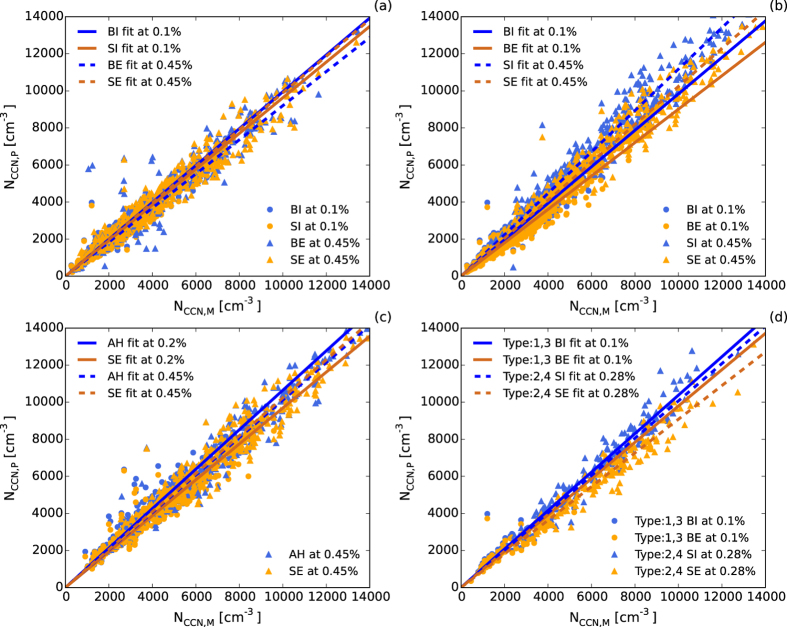
Comparisons of measured and predicted CCN concentrations by (**a**) BI and SI methods at SS of 0.1% and BE and SE methods at SS of 0.45%, (**b**) BI and BE methods at SS of 0.1% and SI and SE methods at SS of 0.45%, (**c**) AH and SE methods at SS of 0.2% and 0.45%, (**d**) BI and BE methods for weather-pollution types 1 and 3 (mist and heavy mist) at SS of 0.1%, and SI and SE methods for weather types 2 and 4 (haze and heavy haze) at SS of 0.28% respectively. The data were fitted by least squares fitting.

Figure 8 shows the comparison of different prediction methods. From Fig. 8a, it can be seen that in general the predictions obtained using the AMS bulk chemical composition method were more accurate than those obtained using the AMS size-resolved chemical composition method under 0.1% supersaturation, while at higher supersaturation (0.45%), the size-resolved chemical composition method was more robust. According to Table 2, the predictions from the size-resolved chemical composition method were closer to observations than those from the bulk chemical composition method when the supersaturation was >0.2% and for weather types from 1 to 6 (different levels of mist and haze included). This indicated that for high supersaturation environments (strong convection weather or over the ocean), the size-resolved chemical composition method is needed for CCN predictions. As shown in Fig. 8b, it was more reliable to assume that aerosols were internally mixed when at low supersaturation (0.1%), due to the external mixing method significantly underestimating the CCN concentration. However, when at high supersaturation (0.45%), the external mixing method was more accurate. This may be because large critical active diameters at low supersaturation corresponding to internally mixed particles, while the critical active diameters at high supersaturation were small, corresponding to fine and mostly externally mixed particles. As summarized in Fig. 8a,b, in general, when at 0.1% supersaturation, the BI method should be used in CCN prediction, and when at high supersaturation, the SE method should be used.

As illustrated in Fig. 8c, at 0.2% supersaturation, the results from the AH method were slightly worse than the SE method, while under higher supersaturation, the AH method was more accurate. From Table 2, it can be seen that the AH was best for all weather episodes. This result demonstrated that under high supersaturation, the chemical method was still unreliable due to the critical diameters of particles at high supersaturation being too small. Figure 8d shows that even at low supersaturation (0.1%) and using the bulk chemical composition method, the predictions from the external mixing method (BE) were better than the internal mixing method (BI) for mist weather (types 1 and 3); however, for haze weather (types 2 and 4), even at relatively high supersaturation (0.28%) and using the size-resolved chemical composition method, the predictions from the internal mixing method (SI) were better. From Table 2, it can be seen that when supersaturation was >0.45%, the SE method was finally better than the SI method, indicating again that in haze episodes, due to the large concentration of highly aged aerosols and particles, the aerosol particles can be considered to be internally mixed for CCN predictions when supersaturation is not relatively low. In the mist episodes, the external mixing assumption was better.

In general, the prediction method using bulk chemical composition was accurate enough under low supersaturation (≤0.1%), and the aerosol external mixing assumption was better in predictions for mist, heavy mist, and fog weather, whereas the internal mixing assumption was applicable to the other weather types.

When the supersaturation was ≥0.2%, the method using size-resolved chemical compositions was better. For hazy and heavy haze weather, the internal mixing assumption was better when SS ≤ 0.28%, while the external mixing assumption was more accurate when SS > 0.28%. For others weather conditions, the external mixing assumption were always better when SS ≥ 0.2%.

### Comparison of CCN activation and prediction with other researches

Some major parameters related to CCN activation under similar supersaturations from various sites of the world are listed in Table 3. Generally, chemical compositions are important to the CCN activation. In relatively clean areas, such as N. America, Europe and S. America, the critical active diameter *D*_c_ is significantly greater than that in polluted areas, such as Asia, exhibiting a possible higher proportion of organic matters and lower hygroscopic ability of aerosols in the clean areas. The largest κ was found in Jeju Island, Korea, showing the influence of sea salts; and the smallest κ was found in Amazonia area in S. America, reflecting a larger fraction of organic aerosols. Correspondingly, in relatively clean areas (N. America, Europe and S. America), the CCN number concentration is far less than those in relatively polluted areas (such as Asian area) (Table 3). This is mainly because the CCN number concentration is closely linked to the aerosol number concentration. In the relatively polluted areas, there are larger numbers of anthropogenic aerosols, which can be easily activated as CCN and interact with cloud processes. By contrast to LinAn’s clean and heavy haze episodes, one can find the κ values were similar, but the activation ratio in heavy haze episode was much higher, showing the effects of aging degree of aerosol on CCN activation ratio. This was also true for sites in Europe, N. America and S. America, where the activation ratio of urban sites with relatively more fresh aerosols was lower than that at rural sites, where the aerosols were relatively aged.

In terms of CCN prediction, most researches in Table 3 have taken aerosol chemical compositions (bulk or size resolved) into consideration; while the mixing state of aerosol particle was often assumed to be internally mixed. In this work, we found that the mixing assumptions (external or internal) and chemical composition information (bulk or size-resolved) should be chosen according to various weather conditions, which can effectively improve the prediction results.

## Conclusions

To investigate the impact of pollution on the CCN activation ability of aerosol particles, a long-term experiment were carried out at LinAn regional GAW station in the YRD area of China. The weather (or pollution) conditions were classified into nine types, representing increasing pollution states from clean to haze and fog. From clean to severe haze pollution (types 0 to 6), the inorganic fraction of aerosol increased, in which nitrate increased significantly, and the mode diameters of the size distribution moved to a larger one, exhibiting the aging of particles. During the experiment, the occurrence of many pollution episodes began with an NPF event, and only under high supersaturation the CCN concentration increased significantly during the growth period of new particles.

The CCN activation curve and its parameters showed different variations for different pollution episodes. In the clean episodes, the aerosol particles were the most internally mixed, had a maximum MAF_f_ at all measured supersaturations with an ideal-shaped activation curve. For pollution with the same level of visibility associated with relative higher RH, the particles were more heterogeneous and externally mixed with lower hygroscopicity and MAF_f_. This may because in the mist and heavy mist weather, due to the abundant water vapor, the internally mixed particles took up water and coagulated at large diameters or grew to droplets, and the rest at investigated diameters were heterogeneous and externally mixed particles; at the same time, heterogeneous reactions probably generated some heterogeneous particles that may also led to this situation; therefore, particles in relatively higher RH conditions exhibited a poor activation ability. In the fog and heavy fog episodes, the aerosols sampled were generally externally mixed particles, with the lowest degree of homogeneity and MAF_f_ of all of the weather types.

The hygroscopic capacity of organic and total inorganic aerosols under nine weather (pollution) types could be classified into two categories: in the mist, heavy mist, fog, heavy fog, and clean episodes, the *κ* of inorganic and organic fractions were ~0.6 and ~0.2, respectively; whereas in the haze, heavy haze, and severe haze episodes, the *κ* of inorganic and organic fractions were ~0.7 and ~0.1, respectively. This result was different from a previous study, and was associated with the variation in aerosol particle chemical composition during different weather in China.

For the time dependent CCN predictions with aerosol size distribution and chemical composition information, the prediction method using bulk chemical composition (all particles have identical chemical composition for the entire size range) was found to be accurate enough under low supersaturation (≤0.1%), while for supersaturation ≥0.2%, the size-resolved chemical composition method was more accurate. As to mixing states, the internal mixing assumption was better in predictions for haze and heavy haze weather conditions when SS ≤0.28%, while for other weather/SS conditions, the external mixing assumption was better. This result was understandable in fog and heavy weather, while in clean weather, it may have resulted from the low concentration and few species of aerosol particles. The experimental average hygroscopicity method was also robust for CCN prediction, especially at high supersaturations, although limited in the YRD due to the limitation of measured κ.

## Methods

### Station and experimental setup

The measurements were performed during January to October 2013 at LinAn regional background station, which is a World Meteorological Organization (WMO) Global Atmosphere Watch regional station (30.3°N, 119.73°E, 138 m a.s.l.) located in the center of the Yangtze River Delta, China^36^. The prevailing winds at LinAn station were northeasterly and southwesterly, accompanied by clean and haze weather.

All instruments were placed in an air-conditioned laboratory with the indoor temperature maintained at 25 °C. An aerosol inlet system through a commercially available PM_10_ impactor (PM_10_ inlet, URG Corporation) was fixed on the rooftop (~5 m a.g.l.). An automatic regenerating adsorption aerosol dryer (Tuch *et al.* 2009) was used with the inlet system to provide low relative humidity (RH) air to ensure that the dried aerosols passed through a splitter via 3/4′′ stainless steel tubes and reached the different instruments. The total sample flow through the dryer inlet was kept at 16.7 lpm to ensure 50% collection efficiency at 10 μm aerodynamic diameter^37^. The dried PM_10_ aerosols (RH < 30%) then reached different instruments for measurements, including an aerodyne aerosol mass spectrometer (AMS) to measure the particle chemical composition, a twin differential mobility particle sizer (TDMPS) to measure the particle number size distributions, and a size-resolved CCN measurement system to obtain the size-resolved CCN activation curves. In addition, a multi-angle absorption photometer (MAAP, model 5012, Thermo Scientific Inc.) was used in parallel to obtain the equivalent mass concentration of black carbon at 637 nm wavelength^38^.

The size-resolved CCN measurement system consisted of a differential mobility analyzer (DMA, TSI 3080), a condensation particle counter (CPC, TSI 3772), and a single-column continuous-flow streamwise thermal-gradient CCN counter (DMT CCNC-100). The DMA was operated at a total flow rate of 1.5 L min^−1^, and the sample flow rates of the CCNC and CPC were 0.5 and 1.0 L min^−1^, respectively. The sample to sheath flow ratio was set to 10 and 9 for the CCNC and DMA, respectively.

For each CCN measurement cycle, five different supersaturations were set: 0.1, 0.2, 0.28, 0.45, and 0.7%. During each supersaturation measurement, 12 different dry aerosol particle diameters were selected by the DMA, ranging from 20 to 300 nm. For each diameter, the CPC and CCNC simultaneously measured the total number of aerosol particles (condensation nuclei, CN) and CCN under the current supersaturation. The measurements took 160 s at each diameter to avoid uncertainty from diameter change, and took 2 min when the supersaturation changed to stabilize the temperature. At 0.1% supersaturation, a longer stable time (6 min) was set when the supersaturation changed from high to low. The whole cycle took ~3 h.

### Calibration

For the supersaturation calibration, the CCNC was calibrated with ammonium sulfate aerosol as described by Rose, *et al.*^39^. The CCNC flow was calibrated according to the CCNC manual. During the experiment, three supersaturation and four flow calibration experiments were performed. For all of the calibrations, high agreement was achieved, with R^2^ values all higher than 0.98.

The particle number size distributions concurrently measured by the TDMPS were used in the CCN multiply charged correction and the total CCN number concentration calculation. The TDMPS calibration and data processing have been described in a previous paper^29^. The aerosol chemical composition size-resolved mass concentration was measured by the AMS; the calibration and data correction for the AMS can also be found in a previous paper^28^.

### CCN activation data fitting

The time series of size-resolved CCN data were first coupled with the CN data measured by the CPC. All data were collected after removing the unstable flow data and abnormal values using the Pauta criterion method. Then the measured CCN data were corrected for multiply charged particles^40^ and the DMA transfer function^39^. After correction, all CCN activation data were fitted with a cumulative Gaussian distribution function (CDF) with three and two parameters as described in a previous paper^11^,^39^.

The midpoint activation diameters (i.e., the critical active diameter) *D*_a_ and *D*_t_, and the CDF standard deviations *σ*_a_ and *σ*_t_ were determined from three-parameter and two-parameter CDF, respectively. The maximum active fraction MAF_f_ was derived from three-parameter CDF. In two-parameter CDF, the MAF_f_ was set to 1 by assuming that all particles were eventually active. *σ*_a_ and *σ*_t_ can be regarded as indicators for the extent of external mixing and the heterogeneity of CCN active particles at ~*D*_a_ and all particles (CCN active and inactive) at ~*D*_t_, respectively. The ratio of the CDF standard deviation and its corresponding critical active diameter can be regarded as an indicator of the degree of heterogeneity of the aerosol particles. More specifically, *σ*_a_/*D*_a_ represents the heterogeneity of the CCN active particle fraction at ~*D*_a_, and *σ*_t_/*D*_t_ represents the overall heterogeneity of both the CCN active and inactive fractions at ~*D*_t_. A detailed description of the CDF and its derived parameters can be found in a previous paper^11^.

### Effective hygroscopicity parameters calculation

The equilibrium supersaturation (SS) of a particle with given diameter (*D*), *κ*, and surface tension of the solution (*σ*_sol_) were determined by the maximum of Equation 1^33^,^39^:


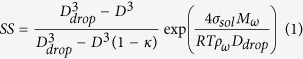


where *D*_*drop*_ is the droplet diameter, *M*_*ω*_ the molecular weight of water, *R* the gas constant, *T* the absolute temperature, *ρ*_*ω*_ the density of water. Therefore, the effective hygroscopicity parameters *κ*_a_ and *κ*_t_ were obtained by using the observed critical diameters (*D*_a_ and *D*_t_) and corresponding supersaturation from Equation 1. κa and κt represent the average hygroscopicity of CCN active particles at ~*D*_a_ and all particles (CCN active and inactive) at ~*D*_t_, respectively.

### CCN prediction

In the CCN prediction, *κ* values derived from the different methods were used to calculate the critical active diameter (*D*_c_) using Equation 1. Then the CCN concentration was obtained by integrating the measured aerosol size distribution above the calculated *D*_c_. For a particle comprising multiple components, *κ* was determined using the simple mixing rule^33^:


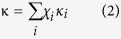


The subscript *i* denotes species, *χ*_*i*_ and *κ*_*i*_ are the volume fraction and corresponding hygroscopic capability of the species, respectively. In the predictions, the values *κ*((NH_4_)_2_SO_4_) = 0.61, *κ*(NH_4_NO_3_) = 0.67, *κ*(black carbon) = 0 were used^20^. The AMS size-resolved data were taken as a 3 h average to improve the signal-to-noise ratio, and the volume of measured species was calculated according to their density^41^,^42^.

After the CCN concentration calculations, all CCN prediction data were fitted by orthogonal distance regression and weighted by inverse measurement uncertainties, for the both measured CCN number concentration (*N*_ccn,m_) and the predicted number concentration (*N*_ccn,p_). A 5% relative error was associated with the fitting. Note that at 0.7% supersaturation, the critical diameters were mainly <50 nm, and the AMS size-resolved mass concentration was unreliable in that size range. Therefore, the CCN prediction methods using size-resolved AMS data were only used when the supersaturation was <0.7%.

## Additional Information

**How to cite this article**: Che, H. C. *et al.* Characterization and parameterization of aerosol cloud condensation nuclei activation under different pollution conditions. *Sci. Rep.* **6**, 24497; doi: 10.1038/srep24497 (2016).

## Figures and Tables

**Table 1:** Classification of different weather corresponding to different extents of aerosol pollution.

Type	Visibility (km)	Relative humidity (%)	Episode	Number of CCN spectra
0	VIS ≥ 10	≤80	Clean	1065
1	5 ≤ VIS < 10	80 < RH ≤ 90	Mist	145
2	5 ≤ VIS < 10	RH ≤ 80	Haze	675
3	1 ≤ VIS < 5	80 < RH ≤ 90	Heavy mist	525
4	1 ≤ VIS < 5	RH ≤ 80	Heavy haze	716
5	VIS < 1	80 < RH ≤ 90	Transition from mist to fog	0
6	VIS < 1	RH ≤ 80	Severe haze	52
7	1 ≤ VIS < 5	RH ≥ 90	Fog	189
8	VIS < 1	RH ≥ 90	Heavy fog	227

**Table 2:** Fitting results of measured and predicted CCN concentrations.

		Type 0	Type 1	Type 2	Type 3	Type 4	Type 6	Type 7	Type 8
0.1%	AH	1.04(0.95)	1.00(0.97)	1.02(0.96)	0.95(0.89)	0.96(0.95)	0.98(0.99)	1.00(0.97)	0.93(0.95)
	BI	0.97(0.96)	1.05(0.97)	0.98(0.97)	1.03(0.91)	0.94(0.98)	0.94(0.99)	1.05(0.98)	1.06(0.98)
	BE	0.91(0.95)	0.99(0.97)	0.85(0.96)	0.98(0.91)	0.83(0.98)	0.84(0.99)	0.99(0.98)	0.99(0.98)
	SI	0.99(0.93)	1.00(0.96)	0.97(0.96)	0.94(0.91)	0.92(0.98)	0.92(0.99)	0.94(0.99)	0.91(0.99)
	SE	1.00(0.93)	1.02(0.94)	0.89(0.96)	0.96(0.89)	0.84(0.98)	0.85(0.99)	0.98(0.98)	0.97(0.98)
0.2%	AH	1.08(0.97)	1.07(0.98)	1.05(0.98)	1.03(0.94)	1.02(0.96)	1.00(0.99)	1.08(0.96)	1.01(0.97)
	BI	1.03(0.95)	1.1(0.99)	1.03(0.99)	1.08(0.94)	1.02(0.98)	1.00(0.99)	1.09(0.97)	1.05(0.98)
	BE	0.97(0.95)	1.04(0.99)	0.90(0.98)	1.03(.094)	0.91(0.98)	0.91(0.99)	1.03(0.97)	0.99(0.99)
	SI	1.09(0.93)	1.07(0.99)	1.01(0.98)	1.03(093)	0.99(0.99)	0.97(0.99)	1.04(0.97)	0.97(0.99)
	SE	1.01(0.95)	1.03(0.99)	0.89(0.98)	1.00(0.93)	0.88(0.99)	0.88(0.99)	1.02(0.97)	0.96(0.99)
0.28%	AH	1.05(0.98)	1.03(0.98)	1.03(0.99)	1.00(0.98)	1.00(0.98)	1.00(0.99)	1.03(0.98)	1.00(0.98)
	BI	1.04(0.97)	1.09(0.99)	1.04(0.99)	1.10(0.98)	1.07(0.98)	1.06(0.99)	1.11(0.97)	1.06(0.99)
	BE	0.99(0.99)	1.04(0.99)	0.93(0.99)	1.05(0.98)	0.96(0.98)	0.96(0.99)	1.05(0.98)	1.01(0.99)
	SI	1.13(0.77)	1.08(0.99)	1.02(0.99)	1.08(0.98)	1.03(0.99)	1.03(0.99)	1.08(0.96)	1.01(0.99)
	SE	1.01(0.96)	1.03(0.99)	0.91(0.98)	1.03(0.98)	0.93(0.98)	0.93(0.99)	1.04(0.97)	1.00(0.99)
0.45%	AH	1.03(0.98)	1.03(0.97)	1.03(0.99)	1.02(0.99)	1.01(0.99)	1.02(0.98)	1.04(0.99)	1.03(0.99)
	BI	1.07(0.97)	1.11(0.99)	1.08(0.98)	1.11(0.99)	1.08(0.99)	1.04(0.99)	1.10(0.98)	1.10(0.99)
	BE	1.02(0.97)	1.08(0.99)	0.98(0.99)	1.07(0.99)	1.00(0.99)	0.97(0.99)	1.07(0.99)	1.06(0.99)
	SI	1.14(0.97)	1.14(0.98)	1.08(0.98)	1.11(0.98)	1.09(0.99)	1.04(0.99)	1.10(0.98)	1.08(0.99)
	SE	1.06(0.98)	1.07(0.99)	0.97(0.98)	1.06(0.99)	0.98(0.99)	0.97(0.99)	1.06(0.98)	1.04(0.99)
0.7%	AH	1.03(0.99)	1.03(0.99)	1.03(0.99)	1.02(0.99)	1.02(0.99)	1.02(0.98)	1.03(0.99)	1.03(0.99)
	BI	1.09(0.98)	1.09(0.11)	1.09(0.97)	1.1(0.94)	1.08(0.99)	1.05(0.99)	1.10(0.97)	1.07(0.99)
	BE	1.05(0.98)	1.06(0.99)	1.01(0.976)	1.07(0.99)	1.02(0.99)	0.98(0.99)	1.07(0.98)	1.05(0.99)
The values are the fitting factors and R^2^ (in brackets).									

**Table 3:** The comparison of CCN activation and prediction in LinAn with other researches.

	Site location	Air mass type	SS (%)	D_c_ (nm)	κ	Nccn (cm^−3^)	Activation ratio	Prediction	
								Chemical composition	Mixing state
This Work	LinAn-clean, China	rural-clean	0.45	60.1 ± 4.4	0.32 ± 0.07	4757 ± 2179	0.46 ± 0.11	bulk	external
	LinAn-heavy haze, China	rural-pollution	0.45	62.0 ± 4.7	0.3 ± 0.07	8697 ± 2692	0.71 ± 0.07	bulk	external
	LinAn-heavy haze, China	rural-pollution	0.28	79.3 ± 5.7	0.34 ± 0.08	7183 ± 2424	0.62 ± 0.06	size-resolved	internal
Asia	Beijing-aged pollution^43^, China	urban-pollution	0.46	59.3 ± 3.0	0.31 ± 0.05	8830 ± 1600	0.74 ± 0.08	—	—
	PRD^11^,^15^, China	urban	0.47	58.3 ± 5.8	0.33 ± 0.08	9760 ± 5320	0.53 ± 0.19	size-resolved	internal
	Hong Kong^19^, China	urban	0.5	56 ± 6.0	0.31 ± 0.10	1815 ± 1285	0.57 ± 0.14	size-resolved	internal
	Kanpur-summer^44^, India	urban	0.5	64.4 ± 11.6	0.24 ± 0.13	5074	0.71	no	—
	Jeju Island^12^, Korea	coastal	0.58	44 ± 3	0.48 ± 0.1	3496 ± 1510	—	bulk	internal
Europe	Vienna^45^, Austria	urban	0.5	—	—	820	0.13	—	—
	Hyytiälä^46^, Finland	rural	0.4	74.82	0.21	—	0.42	—	—
	Vavihill^46^,^47^, Sweden	rural	0.5	—	0.21	1285	0.44	no	—
North America	Colorado^34^, USA	rural	0.36	83.9 ± 7.1	0.18 ± 0.04	—	—	—	—
	Tucson-winter^48^, USA	urban	0.2	—	0.19	420	0.08	no	—
South America	Amazonia^13^, Brazil	remote-clean	0.46	82.8 ± 8.8	0.122 ± 0.04	141 ± 147	0.53 ± 0.13	bulk	internal
	São Paulo^21^, Brazil	urban	0.45	—	0.15 ± 0.04	2202 ± 1035	0.19 ± 0.09	size-resolved	internal
